# Season of birth is associated with birth weight, pubertal timing, adult body size and educational attainment: a UK Biobank study

**DOI:** 10.1016/j.heliyon.2015.e00031

**Published:** 2015-10-12

**Authors:** Felix R. Day, Nita G. Forouhi, Ken K. Ong, John R.B. Perry

**Affiliations:** a MRC Epidemiology Unit, University of Cambridge School of Clinical Medicine, Box 285 Institute of Metabolic Science, Cambridge Biomedical Campus, Cambridge, CB2 0QQ, UK; b Department of Paediatrics, University of Cambridge, UK

**Keywords:** Puberty, Seasonality, Epidemiology, Vitamin D, Sunlight

## Abstract

Season of birth, a marker of *in utero* vitamin D exposure, has been associated with a wide range of health outcomes. Using a dataset of ∼450,000 participants from the UK Biobank study, we aimed to assess the impact of this seasonality on birth weight, age at menarche, adult height and body mass index (BMI). Birth weight, age at menarche and height, but not BMI, were highly significantly associated with season of birth. Individuals born in summer (June–July–August) had higher mean birth weight (P = 8 × 10^−10^), later pubertal development (P = 1.1 × 10^−45^) and taller adult height (P = 6.5 × 10^−9^) compared to those born in all other seasons. Concordantly, those born in winter (December–January–February) showed directionally opposite differences in these outcomes. A secondary comparison of the extreme differences between months revealed higher odds ratios [95% confidence intervals (CI)] for low birth weight in February vs. September (1.23 [1.15–1.32], P = 4.4 × 10^−10^), for early puberty in September vs. July (1.22 [1.16–1.28], P = 7.3 × 10^−15^) and for short stature in December vs. June (1.09 [1.03–1.17], P = 0.006). The above associations were also seen with total hours of sunshine during the second trimester, but not during the first three months after birth. Additional associations were observed with educational attainment; individuals born in autumn vs. summer were more likely to continue in education post age 16 years (P = 1.1 × 10^−91^) or attain a degree-level qualification (P = 4 × 10^−7^). However, unlike other outcomes, an abrupt difference was seen between those born in August vs. September, which flank the start of the school year. Our findings provide support for the ‘fetal programming’ hypothesis, refining and extending the impact that season of birth has on childhood growth and development. Whilst other mechanisms may contribute to these associations, these findings are consistent with a possible role of *in utero* vitamin D exposure.

## 1 Introduction

Several studies have reported associations between month, or season, of birth and risks of later life health outcomes. The most compelling associations to date appear to be those with immune-related disease (ID) [1], such as type 1 diabetes [2] and multiple sclerosis [3]. Other associations have been reported with diverse health outcomes, including cardiovascular disease [4], type 2 diabetes [5], psychiatric disorders [6] and all-cause mortality [7]. The most comprehensive assessment to date performed a “phenome-wide” scan in the health records of over 1.7 million US individuals, identifying 55 robust disease associations [8]. Another large analysis of the Kadoorie Biobank study [9] reported robust associations between season of birth and adult adiposity. That analysis of ∼500,000 participants from 10 geographically diverse areas of China highlighted increased adult BMI and waist circumference in individuals born in March–July, and shorter leg lengths for those born in February–August. Season of birth associations therefore provide direct support for the ‘fetal origins of adult disease hypothesis’ that intra-uterine exposures (independent of genetic effects) may have long-term impacts on later health [10].

Various mechanisms have been suggested to underlie month of birth associations, including seasonal differences in maternal exposure to meteorological factors (such as sunshine), air pollution, food supply, diet and physical activity [4]. Marked seasonal changes have been reported in maternal circulating 25-hydroxyvitamin D levels (25(OH)D) [11], which reflect sunshine exposure and directly influence fetal vitamin D exposure. Hence, newborn circulating 25-hydroxyvitamin D_3_ levels also vary markedly by season of birth, with almost two-fold higher levels in summer compared to winter births reported in a Danish population study [12].

Under the hypothesis that season of birth associations are primarily driven by changes to circulating 25(OH)D, we prioritised a previously untested trait for month of birth effects – puberty timing. Age at menarche is a well-recalled measure of pubertal timing in girls and has been linked to vitamin D status in prospective [13] and genetic [14] studies. Furthermore, we extended these analyses to assess the role of birth weight, height and BMI as potential confounders/mediators of this association.

In up to 452,399 white UK Biobank participants born in the UK and Ireland, we identify robust associations between season of birth and early life growth and development.

## 2 Methods

### 2.1 Population and study design

The UK Biobank study design has been previously reported [15] [16]. Briefly, all people aged 40–69 years who were registered with the National Health Service and living up to ∼25 miles from one of the 22 study assessment centres were invited to participate in 2006–10. Overall, about 9.2 million invitations were mailed in order to recruit 503,325 participants (i.e. a response rate of 5.47%). Extensive self-reported baseline data were collected by questionnaire, in addition to anthropometric assessments. For the current analysis, individuals of non-white ancestry or born outside of the United Kingdom and Republic of Ireland were excluded from analysis to reduce heterogeneity in maternal exposure. All participants provided informed written consent, the study was approved by the National Research Ethics Service Committee North West – Haydock, and all study procedures were performed in accordance with the World Medical Association Declaration of Helsinki ethical principles for medical research.

### 2.2 Exposures and outcomes

Our primary exposure of interest was season of birth, which was based on month of birth recorded in all study participants by questionnaire. We categorised the month of birth into seasons, defined as Spring (March–April–May), Summer (June–July–August), Autumn (September–October–November) and Winter (December–January–February). The primary outcomes of interest were the participants' birth weight, their height/BMI at recruitment, and among women, their age at menarche. Birth weight was recalled by questionnaire and reported in kilograms. Birth weight was treated both as a continuous quantitative trait and a case-control outcome, with weights below 1 Kg and above 6 Kg (approximating 4 standard deviations from the mean) excluded from analysis. Low birth weight cases were defined as < 2.5 Kg, controls were all birth weights >= 2.5 Kg. Age at menarche in women was self-reported in whole years, and women with a reported age < 8 or > 19 were excluded as outliers. Early menarche was defined as 8–11 years inclusive. Body mass index (Kg/m^2^) and height in centimetres were measured at the assessment centre and treated as continuous outcomes, excluding individuals >4 SDs from the mean. A short stature case-control variable was additionally defined as the bottom 5% of individuals (within sex) vs all others.

We estimated maternal sunshine exposure using recorded data from the Met Office (http://www.metoffice.gov.uk/pub/data/weather/uk/climate/datasets/Sunshine/date/UK.txt). For each individual, we calculated the cumulative hours of sunshine recorded for each month averaged across the UK in the 9 months preceding their birth month and the 3 months after. These were then grouped into four groups – three trimesters (including the month of birth) and the first 3 months after their birth month.

Secondary analyses were performed across past or current diseases self-reported in response to the question “Has a doctor ever told you that you have had any of the following conditions? (You can select more than one answer)”. To ensure good discrimination between medical conditions, the data were collected using a computer-assisted personal interview (CAPI), administered by trained interviewers. To provide sufficient statistical power we considered only those diseases/outcomes with least 500 cases in either sex (∼0.2% prevalence). In total, we considered 185 diseases or health outcomes which we previously defined and tested [17]. This led to a conservative multiple testing corrected P-value of 6.8 × 10^−5^ (0.05 / 185 traits × 4 seasons) for this untargeted analysis.

Three educational attainment variables were created in response to the touch-screen questionnaire completed by participants. Individuals who responded “prefer not to answer” were set to missing. Individuals who held a college or university degree (‘yes’ coded as a case, ‘no’ and non-missing as control), the age at completion of full-time education (cases defined as > 16 years, controls as <= 16), and thirdly individuals reporting no listed qualifications (cases defined as holding none, controls as one or more qualifications).

### 2.3 Statistical analysis

Birth month and birth season variables were coded ‘1′ for the month/season of interest and ‘0′ for all others. Linear regression models were performed to test the association between birth season and each of our four primary outcomes (self-reported birth weight and age at menarche, and current measured BMI and height). Prior to analysis, birth weight and estimated sunshine exposure were inverse-normally transformed to have a mean = 0 and SD = 1. Our significance threshold was set at P-value (0.05 / (4 traits × 4 seasons)) = 0.003 to declare a birth season effect. To ascertain the shape of any resulting associations, we additionally repeated the analyses using individual birth months as the exposure (e.g January births vs all others). All models were adjusted for age, sex (where appropriate) and socio economic position (SEP) defined by 11 principal components (PC) explaining > 99% of the trait variance [17]. Variables included in this PC construction included alcohol intake, educational attainment, participant and maternal smoking, household income, and Townsend index of material deprivation based on geographical location of residence. Analyses of educational attainment were adjusted only for age and sex. Low birth weight, in addition to other self-reported disease cases or adverse health outcomes were analysed in a logistic regression framework with the same covariates.

## 3 Results

255,769 individuals (100,128 men, 155,641 women) had a self-reported birth weight > 1 kg, 9.8% (N = 25,054) of whom reported low birth weight (< 2.5 Kg). Age at menarche between the ages of 8 and 19 years inclusive was self-reported in 238,014 women. Height and BMI measurements were available in 451,435 and 452,399 individuals, respectively after exclusions and covariate adjustments.

### 3.1 Season of birth and birth weight

**Fig. 1:**
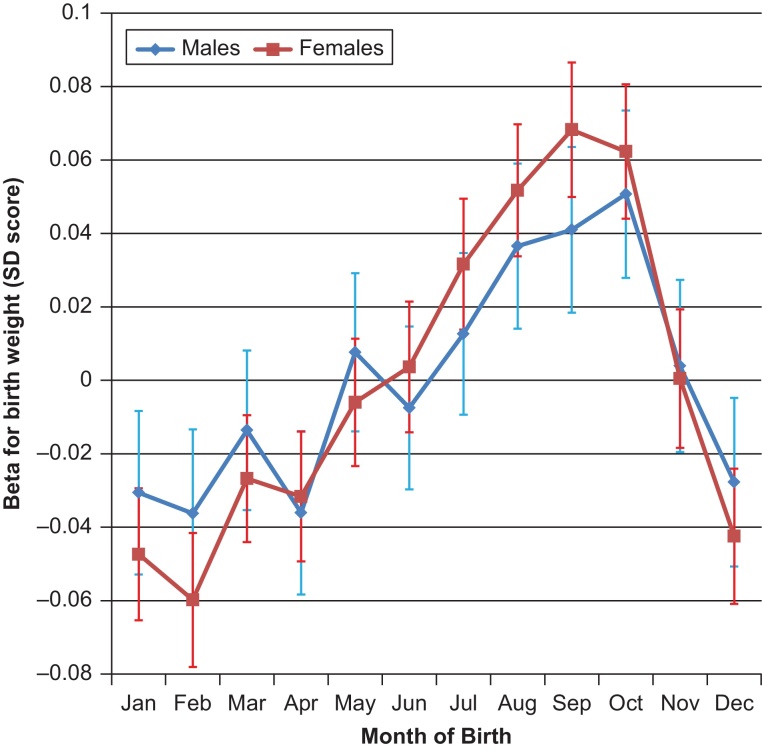
Month of birth associations with birth weight in the UK Biobank study. The Y-axis indicates regression coefficients (95% CI) for the association of each birth month (vs. all other 11 months) on birth weight SD score.

Season of birth was associated with birth weight (as a continuous trait); each of the four seasons showed significant differences to the other 3 seasons (Table 1). Effect estimates ranged from +0.05 SDs for autumn births (vs. the other 3 seasons: P = 5.6 × 10^−25^, N = 255,769) to −0.05 SDs for winter births (P = 3.8 × 10^−29^), with significant heterogeneity between sexes (summer and winter P_het_ < 0.05). Associations with month of birth varied continuously throughout the year (Fig. 1), with a peak in September (vs. the other 11 months: +0.06 SDs, P = 2.1 × 10^−15^) and a trough in February (−0.05 SDs, P = 5.3 × 10^−12^). Associations with the dichotomised trait, low birth weight (case N = 25,054), showed similar patterns (Table 2). Individuals born in February were more likely to have low birth weight than those born in September (OR 1.23 [1.15–1.32], P = 4.4 × 10^−10^), an effect which was significantly different between sexes (P_Het_ = 0.02).

### 3.2 Season of birth and pubertal timing in women

**Fig. 2:**
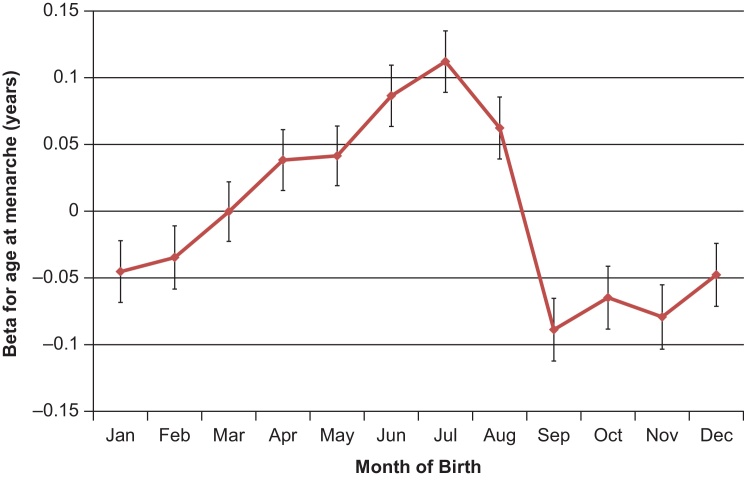
Month of birth associations with age at menarche in the UK Biobank study. The Y-axis indicates regression coefficients (95% CI) for the association of each birth month (vs. all other 11 months) on age at menarche.

Season of birth was associated with reported age at menarche in women; each of the four seasons showed significant differences to the other 3 seasons (Table 1). Effect estimates ranged from +0.11 years for summer births (vs. the other 3 seasons: P = 1.1 × 10^−45^, N = 238,014) to −0.09 years for autumn births (P = 2.5 × 10^−34^). Associations with month of birth varied continuously throughout the year (Fig. 2), with a peak in July (vs. the other 11 months): +0.11 years, P = 1.4 × 10^−21^) and trough in September (−0.09, P = 1.2 × 10^−13^). At the monthly extremes, individuals born in September were ∼20% more likely to enter puberty early (age 8–11, 48,314 cases) than those born in July (OR 1.22 (1.16–1.28), P = 7.3 × 10^−15^). These associations appeared independent of birth weight (Table 1).

### 3.3 Season of birth and adult body size

**Fig. 3:**
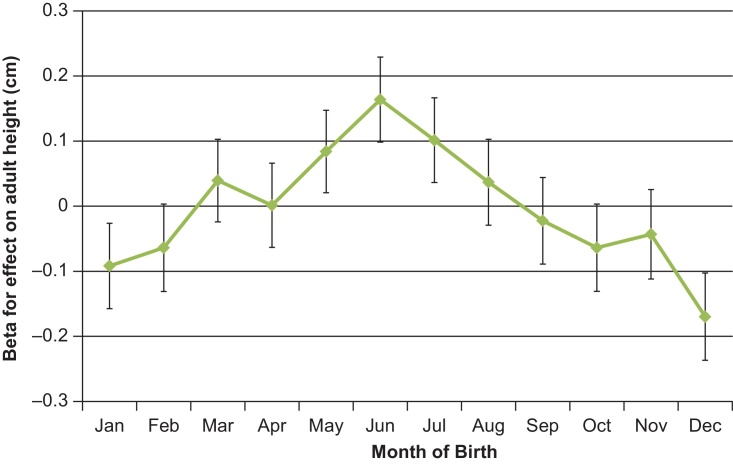
Month of birth associations with adult height in the UK Biobank study. The Y-axis indicates regression coefficients (95% CI) for the association of each birth month (vs. all other 11 months) on adult height.

Season of birth was associated with adult height, but not adult BMI (Table 1, Fig. 3). Effect estimates on adult height ranged from +0.12 cm for summer births (vs. the other 3 seasons: N = 451,435, P = 6.5 × 10^−9^) to −0.13 cm for winter births (P = 1.2 × 10^−9^). Peak month of birth differences were seen between June vs. December: +0.31 cm taller height (P = 2.0 × 10^−11^) and lower risk of short stature (OR 0.92 [0.86–0.97], P = 6 × 10^−3^). Among women, adjustment for age at menarche and birth weight attenuated the association between winter births and shorter adult height, but did not attenuate the association between summer births and taller adult height, and augmented the association between autumn births and shorter adult height (Table 1).

### 3.4 Associations with estimated sunshine exposure

To test the putative effects of antenatal sunshine exposure, we estimated each participant's sunshine exposure during each trimester of pregnancy using meteorological data on monthly total hours of sunshine in the UK, available from the UK Met Office (see methods). As expected, estimated sunshine exposure during the first trimester was strongly correlated with summer (r = −0.73) and winter births (r = 0.68), second trimester with spring (r = −0.74) and autumn (r = 0.67) births and third trimester with summer (r = 0.68) and winter (r = −0.72) births. Assessment of the three traits with significant seasonal effects demonstrated estimated sunshine exposure associations concordant with the observed season of birth associations (Table 3). For each trait, estimated sunshine exposure during the second trimester appeared most significant, with additional third trimester effects for birth weight and height, and first trimester associations for menarche (Table 3). No association was observed with estimated sunshine exposure during the first 3 months after birth (P > 0.05).

### 3.5 Associations with other outcomes

**Fig. 4:**
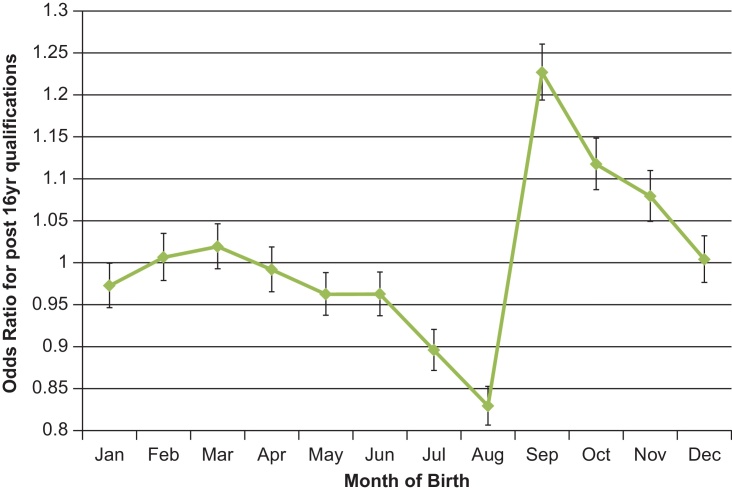
Month of birth associations with education post-16 years old in the UK Biobank study. The Y-axis indicates odds ratio (95% CI) for the association of each birth month (vs. all other 11 months) on the odds of continuing in full-time education post-16 years old.

To assess the potential impacts of the season of birth associations on later health and other outcomes, we systematically tested associations between season of birth and a broad range of 185 disease outcomes. After correction for multiple testing (corrected P-value threshold < 6.8 × 10^−5^), no disease association was seen with any season of birth. We next assessed whether any of the covariates included in these models (age, sex and socio-economic position) was associated with season of birth. As expected, age and sex were not associated with season of birth (all P > 0.05), however several principal components of socio-economic position were (P_min_ = 4 × 10^−34^). These associations were driven by a primary effect of season of birth on educational attainment (Table 4). Individuals born in autumn were more likely to continue in education post age 16 years (vs. the other 3 seasons: OR 1.17 [1.15–1.19], P = 3.8 × 10^−79^). The pattern of association between month of birth and educational attainment differed strikingly to those with birth weight, age at menarche and adult height, with an abrupt contrast between individuals born in September vs. August (education post-16: OR 1.43 [1.38–1.48]) (Fig. 4). Furthermore, the difference between autumn vs. summer births was significantly larger in men (education post-16: OR 1.30 [1.26–1.34]) than in women (1.21 [1.18–1.25], P_Het_ = 0.003).

## 4 Discussion

In this large study of ∼500,000 UK individuals, we describe the most comprehensive assessment to date of the impact of birth season on childhood growth and physical development. In support of several other studies [18] [19], we identify highly significant seasonal changes in birth weight. This was represented by higher birth weights for those born in autumn, alongside lower birth weights for those born in winter. Concordant effects were seen on the risk of low birth weight, and estimates were significantly larger in women than in men. Extensive evidence from randomised controlled trials [20] support maternal 25(OH) vitamin D as the causal mechanism. However, given the variability of results reported in other studies assessing seasonality and birth weight [18] [21], it is likely that additional mechanisms specific to certain environments may also play a role, yet the physiological processes behind the resulting impact on birth weight remain unclear. Vitamin D is important for bone development and may act as a rate-limiting factor for growth.

Seasonality in childhood growth has long been described. Humans, and also animals, show fastest growth in spring and summer and slowest growth in autumn and winter [22]. Our findings extend this by demonstrating robustly for the first time an association between season of birth and puberty timing in girls. Although this association was independent of birth weight, the similar pattern of month of birth associations suggests a common mechanism. Although the possible mechanisms are more speculative, circulating levels of 25(OH)D in children have been prospectively linked to puberty timing [13]. Furthermore, recent genetic studies have indicated potential aetiological roles for the vitamin D receptor (VDR) and related nuclear hormone receptors in pubertal timing [14]. Our observed season of birth effects on puberty timing partly explained our downstream association between season of birth with adult height, but did not attenuate the summer or autumn effects on height.

The lack season of birth associations observed here for adult BMI appear discordant to those recently reported by the Kadoorie Biobank study [9], however in that paper the authors noted substantial variability by geographic region in China. In contrast, we saw no association between month of birth and BMI in a relatively smaller geographic area. In a small study higher newborn 25(OH)D_3_ levels were reportedly associated with higher risk of adult overweight [23], however our null finding for BMI is supported by the reported null association between genetically-predicted 25(OH)D levels and adult BMI [24]. However, these findings collectively suggest that multiple mechanisms may mediate observed month of birth associations, some of which might be specific to geography and environment. This is further illustrated by the association between month of birth and educational attainment. This strong association, centred on the striking gap between August vs September births, is well documented [25] and is explained by school entry policy. In the UK, school entry occurs annually in September; eligible children are those who reach school age by end of August. Hence, children born in September are almost one year older than their classmates born in August. This leads to variation in physical and academic performance within each school year. We demonstrate that this variation extends to the duration of full-time education and the likelihood of achieving qualifications.

Collectively our findings support the existing season of birth literature, refining and extending the impact this has on childhood growth and development. This provides direct support for the ‘fetal origins of adult disease hypothesis' that intra-uterine exposures impact health outcomes many years later [10]. Analyses of estimated sunshine exposure during maternal pregnancy indicated that the 2nd trimester was likely the key time for these exposures. Furthermore, the lack of association with estimated sunshine exposure during the first 3 postnatal months indicates that the effect is ‘programmed’ *in utero*. It remains unclear how these effects are programmed and what physiological mechanisms make them act years after the exposure. Through systematic assessment of almost 200 health/disease traits, we were able to eliminate any large effects of birth seasonality on many common diseases in the UK population. Due to the conservative multiple-test correction thresholds, it still remains possible however that season of birth may have modest effects on other previously unsuspected health outcomes, as seen in other populations [8].

Month of birth is highly likely to be randomised to confounding factors, and resulting associations are not subject to reverse causality. These associations therefore represent causal, rather than correlative, relationships with effect sizes similar to genetic determinants identified for these traits [14] [26] [27]. While genetic factors are unlikely to contribute to the current associations with birth month, future identification of possible genetic interactions with birth month may help to inform the mechanisms involved. The other strengths of our study include a large sample size of individuals without biased ascertainment for birth, alongside broad clinical phenotyping. Limitations of the study include no direct measurement of maternal/fetal vitamin D status to fully establish a causal mechanism. Self-reported variables may be inaccurate or subject to recall bias and no quantitative puberty measure was available in men. Previous studies have however noted accurate recall of birth weight [28] and menarche age [29] in later life, including assessment within UK Biobank [28]. Information on parents’ socioeconomic status was not available. Similarly, no information was available on maternal residential location, individually-measured sunshine exposure, or vitamin D supplementation during pregnancy, however even the youngest participant in the UK Biobank (aged 38 in our analysis) was conceived during a time when gestational vitamin D supplementation was not recommended in the UK. We anticipate that all of these issues would impact the false-negative rate of our study, rather than the validity of our current findings.

In summary, we provide robust evidence linking season of birth to childhood growth and development, in addition to confirming the known associations of timing of birth and educational attainment. While the associations between season of birth, or estimated antenatal sunshine exposure, with birth weight are consistent with experimental effects of *in utero* vitamin D exposure on fetal growth, differing patterns of seasonality and independent associations suggest that other mechanisms may link season of birth to adult height and also puberty timing in women. Future work should aim to better understand the mechanisms linking *in utero* exposures to outcomes years later in life.

## Declarations

### Author contribution statement

Felix Day, John Perry: Conceived and designed the experiments; Performed the experiments; Analyzed and interpreted the data; Wrote the paper.

Ken Ong: Conceived and designed the experiments; Analyzed and interpreted the data; Wrote the paper.

Nita Forouhi: Conceived and designed the experiments; Analyzed and interpreted the data.

### Funding statement

This work was supported by the Medical Research Council (Unit Programme number MC_UU_12015/2).

### Competing interest statement

The authors declare no conflict of interest.

### Additional information

No additional information is available for this paper.

## Acknowledgements

This research has been conducted using the UK Biobank Resource. Data associated with this study is available via application to UK biobank at http://www.ukbiobank.ac.uk.

## Figures and Tables

**Table 1:** Season of birth associations with birth weight, adult height and BMI in men and women, and age at menarche in women: UK Biobank study (max N: 452,399).

		Spring		Summer		Autumn		Winter	
	Sample N	Beta (SE)	P	Beta (SE)	P	Beta (SE)	P	Beta (SE)	P
Birth weight (SDs)									
All	255769	−0.02 (0.004)	3.5E–07	0.03 (0.005)#	8.0E–10	0.05 (0.005)	5.6E–25	−0.05 (0.005)#	3.8E–29
Men	100128	−0.02 (0.007)	1.9E–02	0.02 (0.007)	2.0E–02	0.04 (0.007)	1.3E–07	−0.04 (0.007)	2.1E–07
Women	155641	−0.03 (0.006)	3.5E–06	0.04 (0.006)	1.2E–09	0.05 (0.006)	2.7E–19	−0.06 (0.006)	1.2E–24
Age at Menarche (years)									
Women	238014	0.03 (0.007)	9.4E–06	0.11 (0.008)	1.1E–45	−0.09 (0.008)	2.5E–34	−0.05 (0.008)	1.0E–11
+ adj Birthweight (BW)	152477	0.04 (0.009)	1.2E–04	0.09 (0.009)	3.3E–24	−0.09 (0.01)	7.1E–20	−0.05 (0.009)	1.6E–07
Adult Height (cm)									
All	451435	0.05 (0.021)	1.2E–02	0.12 (0.021)	6.5E–09	−0.05 (0.022)	1.7E–02	−0.13 (0.022)	1.2E–09
+ adj BW	255160	0.05 (0.027)	9.5E–02	0.1 (0.028)	1.6E–04	−0.11 (0.028)	8.4E–05	−0.05 (0.028)	9.7E–02
Men	206995	0.09 (0.032)	4.0E–03	0.13 (0.033)	1.2E–04	−0.08 (0.034)	1.5E–02	−0.15 (0.033)	6.7E–06
Women	244344	0.02 (0.027)	5.1E–01	0.11 (0.028)	3.8E–05	−0.02 (0.028)	4.0E–01	−0.11 (0.028)	6.1E–05
+ adj Menarche	237259	0.01 (0.028)	7.0E–01	0.07 (0.028)	1.0E–02	0.01 (0.029)	7.7E–01	−0.09 (0.028)	1.0E–03
+ adj BW & Menarche	152106	0.01 (0.034)	7.7E–01	0.07 (0.034)	5.1E–02	−0.07 (0.035)	5.5E–02	−0.01 (0.035)	7.0E–01
Adult BMI (kg/m2)									
All	452,399	0.02 (0.015)	2.4E–01	0 (0.016)	8.7E–01	−0.03 (0.016)	6.8E–02	0.01 (0.016)	4.4E–01

Spring (March–April–May), Summer (June–July–August), Autumn (September–October–November) and Winter (December–January–February).

# Denotes significant heterogeneity (I^2^ P < 0.05) between men and women.

**Table 2:** Season of birth associations with low birth weight (< 2.5 Kg) in the UK Biobank study.

	All (N = 25,054 cases)		Men (N = 7,072 cases)		Women (N = 17,982 cases)	
	OR (95% CI)	P	OR (95% CI)	P	OR (95% CI)	P
Spring	1.03 (1.00–1.06)	5.0E–02	1.00 (0.95–1.06)	9.3E–01	1.04 (1.01–1.08)	2.4E–02
Summer	0.90 (0.88–0.93)	7.0E–11	0.91 (0.86–0.96)	1.0E–03	0.90 (0.87–0.93)	1.7E–08
Autumn	0.92 (0.89–0.95)	1.8E–07	0.97 (0.91–1.02)	2.6E–01	0.90 (0.87–0.93)	4.0E–08
Winter	1.16 (1.13–1.19)	1.7E–22	1.13 (1.07–1.19)	1.3E–05	1.17 (1.13–1.21)	1.3E–18
Feb vs Sept	1.23 (1.15–1.32)	4.4E–10	1.13 (1.00–1.28)	4.5E–02	1.28 (1.18–1.38)	9.4E–10

Spring (March–April–May), Summer (June–July–August), Autumn (September–October–November) and Winter (December–January–February).

**Table 3:** Antenatal and early postnatal estimated sunshine exposure associated with birth weight, age at menarche and adult height, in the UK Biobank study.

	Birthweight		Age at Menarche		Adult Height	
	Effect (SE)	P	Effect (SE)	P	Effect (SE)	P
1st Trimester	−0.01 (0.003)	4.0E–02	−0.04 (0.006)	3.3E–13	−0.01 (0.016)	6.5E–01
2nd Trimester	0.02 (0.002)	5.3E–23	−0.03 (0.003)	6.2E–26	−0.04 (0.009)	1.2E–04
3rd Trimester	0.02 (0.003)	2.5E–07	0.00 (0.006)	7.8E–01	0.05 (0.016)	1.0E–03
Total Antenatal	0.02 (0.002)	2.2E–17	−0.03 (0.003)	9.0E–22	−0.03 (0.009)	1.0E–03
Postnatal 3 months	0.00 (0.003)	3.8E–01	−0.01 (0.006)	2.8E–01	0.02 (0.016)	1.8E–01

Estimated sunshine exposure in each trimester was adjusted for sunshine exposure in the other two trimesters in a joint model. ‘Total Antenatal’ is the sum of the three trimesters. Postnatal 3 months sunshine exposure was adjusted for the three trimesters. Effect estimates indicate the effect of a + 1 SD increase in estimated sunshine exposure on birth weight or height Z-scores, or on age at menarche in years.

**Table 4:** Season of birth associated with educational attainment in the UK Biobank study.

	Degree-level		Education post age 16 years		No Qualifications	
Month of Birth	OR [95% CI]	P	OR [95% CI]	P	OR [95% CI]	P
Spring	1.02 [1.01–1.03]	7.0E–03	0.99 [0.97–1.0]	1.9E–01	0.96 [0.95–0.98]	8.1E–05
Summer	0.96 [0.94–0.97]	5.2E–09	0.87 [0.86–0.89]	1.9E–52	1.03 [1.01–1.05]	1.0E–03
Autumn	1.02 [1.01–1.03]	1.4E–02	1.17 [1.15–1.19]	3.8E–71	1.01 [0.99–1.03]	1.3E–01
Winter	1.0 [0.99–1.02]	5.0E–01	0.99 [0.98–1.01]	4.0E–01	0.99 [0.98–1.01]	5.0E–01
Autumn vs Summer	1.05 [1.03–1.07]	4.0E–07	1.25 [1.22–1.28]	1.1E–91	0.99 [0.97–1.01]	3.4E–01
September vs. August	1.10 [1.06–1.13]	1.9E–08	1.43 [1.38–1.48]	2.9E–79	0.95 [0.91–0.99]	7.0E–03

Spring (March–April–May), Summer (June–July–August), Autumn (September–October–November) and Winter (December–January–February). N = 139,153 individuals with a university degree-level qualification were compared to N = 306,960 controls. N = 106,876 individuals who continued in full time education beyond age 16 years old were compared to N = 200,860 controls who left education at <= 16 years. N = 79,763 individuals with no reported qualifications were compared to N = 366,350 controls.
